# Environment Still Matters: Examining Person-Place-Relationships in the Old and the New Normal Across Settings

**DOI:** 10.1093/geroni/igab046.2033

**Published:** 2021-12-17

**Authors:** Frank Oswald, Habib Chaudhury, Amanda Grenier

## Abstract

In environmental gerontology, the home and the neighborhood have always been of particular interest for empirical research. Issues such as orientation and safety, place attachment and biographical bonding, have proven to be important for community dwellings older adults and for those living in care homes. However, with Covid-19, the seemingly stable person-place-relationships have been challenged. This symposium provides a set of applied research contributions that demonstrate the persistent salience of the environment by examining person-place-relationships in the old and the new normal in private homes and care homes. Contributions draw from ideas of “precarious ageing” (Grenier & Phillipson) and “pandemic precarity”, for instance to understand housing insecurity, while concepts from environmental gerontology are used to explain processes of environmental agency and belonging. The first contribution by Mahmood and colleagues introduces an environmental audit tool for people at risk of homelessness to assess built environmental features of housing and neighborhood that support housing stability in the face of insecurity. Second, Wanka provides data from people framed as ‘risk-groups’ through the Covid-19 pandemic and how they dealt with contact restrictions, showing the role of intergenerational neighborhood relations to mediate risks of pandemic precariousness. Third, Elkes examined mobility and wayfinding challenges for residents in a long-term care home and subsequent environmental interventions to improve orientation. Forth, Leontowitsch and colleagues present findings from long-term care home residents during the pandemic to gain understanding of their experiences of social isolation and a biographical sense of resilience. Finally, Amanda Grenier will serve as the session’s discussant.

